# Downregulation of the lncRNA ASB16-AS1 Decreases LARP1 Expression and Promotes Clear Cell Renal Cell Carcinoma Progression *via* miR-185-5p/miR-214-3p

**DOI:** 10.3389/fonc.2020.617105

**Published:** 2021-02-19

**Authors:** Mingzi Li, Bingde Yin, Mulin Chen, Jingtao Peng, Xinyu Mu, Zhen Deng, Jiantao Xiao, Weiguo Li, Jie Fan

**Affiliations:** ^1^ Department of Urology, Shanghai General Hospital, School of Medicine, Shanghai Jiaotong University, Shanghai, China; ^2^ Department of Urology, Minhang Hospital, Fudan University, Shanghai, China; ^3^ Department of Urology, Union Hospital, Tongji Medical College, Huazhong University of Science and Technology, Wuhan, China; ^4^ Department of Urology, Zhongnan Hospital of Wuhan University, Wuhan, China

**Keywords:** long non-coding RNA, lncRNA ASB16-AS1, miR-185-5p, miR-214-3p, LARP1, clear cell renal cell carcinoma

## Abstract

Clear cell renal cell carcinoma (ccRCC) comprises approximately 75% of renal cell carcinomas, which is one of the most common and lethal urologic cancers, with poor quality of life for patients and is a huge economic burden to health care systems. It is imperative we find novel prognostic and therapeutic targets for ccRCC clinical intervention. In this study, we found that the expression of the long noncoding RNA (lncRNA) ASB16-AS1 was downregulated in ccRCC tissues compared with non-diseased tissues and was also associated with advanced tumor stage and larger tumors. By constructing cell and mouse models, it was found that downregulated lncRNA ASB16-AS1 enhanced cell proliferation, migration, invasion, and promoted tumor growth and metastasis. Furthermore, by performing bioinformatics analysis, biotinylated RNA pull-downs, AGO2-RIP, and luciferase reporter assays, our findings showed that downregulated ASB16-AS1 decreased La-related protein 1 (LARP1) expression by inhibiting miR-185-5p and miR-214-3p. Furthermore, it was found that overexpression of LARP1 reversed the promotive effects of downregulated ASB16-AS1 on ccRCC cellular progression. Our results revealed that downregulated ASB16-AS1 promotes ccRCC progression *via* a miR-185-5p-miR-214-3p-LARP1 pathway. We suggest that this pathway could be used to monitor prognosis and presents therapeutic targets for ccRCC clinical management.

## Introduction

Renal cell carcinoma (RCC) is one of the most common types of urologic cancers accounting for more than 90% of renal malignancies (1, 2). Clear cell renal cell carcinoma (ccRCC) comprises approximately 75% of RCCs and is the most lethal pathological subtype of RCC (3). More than 30% of ccRCC patients are diagnosed with metastasized disease and have a 13-month median survival time (4). There are many risk factors such as dietary habits, occupational exposure, and physical inactivity that can lead to ccRCC tumorigeneses. The inefficiency of treatment and limitations in diagnosing ccRCC contribute to the poor quality of life for patients and the huge economic burden this disease has on health care systems (5, 6). Therefore, it is imperative we find novel diagnostic and therapeutic targets for clinical management and intervention.

Long noncoding RNAs (lncRNAs) are one type of noncoding RNA consisting of more than 200 nucleotides (7). The underlying molecular mechanisms of lncRNA have been revealed in the past decades. For example, LncRNAs can act as a molecular sponge for microRNAs (miRs) (8) or interact with proteins, modulating their functions (9). LncRNAs are involved in many biological functions, especially tumorigenesis (10–12). Recently, the roles of lncRNAs in ccRCC tumorigenesis have been partly demonstrated; He et al. found that MEG3 regulates ccRCC progression *via* sponging miR-7 (13); Yang FQ et al. investigated the role of HOXA11-AS in ccRCC progression *via* promoting ccRCC growth and invasion ability (14); Qi Y et al. demonstrated that PENG suppresses ccRCC proliferation *via* sponging miR-15b (15); and the tumor-suppressing role of HOTAIRM1 in ccRCC has recently been demonstrated (16). These studies suggest that lncRNAs play essential roles in ccRCC development.

ASB16-AS1 is localized to 17q21, and is approximately 2275 bp. Previous studies have reported that lncRNA ASB16-AS1 functions as a microRNA sponge and regulates cell proliferation, migration, invasion, and apoptosis in several cancers including hepatocellular carcinoma, glioma, non-small lung cancer, and cervical cancer (17–20), indicating that ASB16-AS1 play its crucial role in tumorigenesis. However, whether ASB16-AS1 exerts its function in ccRCC progression is poorly understood.

We hypothesized that ASB16-AS1 is involved in ccRCC progression and regulates ccRCC cell functions. Firstly, we tested ASB16-AS1 expression in ccRCC tissues. We then constructed *in vitro* and *in vivo* models to demonstrate the biological functions of ASB16-AS1 in ccRCC. Furthermore, we conducted bioinformatic analysis, AGO2-RIP, biotinylated RNA pull-downs, and luciferase reporter assays to elucidate the underlying molecular mechanisms of ASB16-AS1. Collectively, our data suggest that ASB16-AS1 could be used to monitor prognosis and presents therapeutic targets, altogether providing new insights regarding ccRCC basic research.

## Materials and Methods

### Clinical Samples

ccRCC tumors and adjacent non-diseased tissues were collected from 42 patients with ccRCC who received operative treatment at the Department of Urology, Shanghai General Hospital between August 2012 to December 2013. The specimens were collected from a tumor and a region at least 5 cm away from the tumor in each patient. The histological diagnosis was confirmed by two pathologists using hematoxylin and eosin stained sections. Following the American Joint Committee on Cancer (AJCC) guidelines, the pathological stage of each tumor was also determined by two pathologists. All patients provided informed consent.

### Cell Culture and Transfection

The ccRCC cell lines A498, 786-O, 769-P, CAKI-1, OS-RC-2, ACHN, and the human kidney proximal tubular epithelial cell line, HK-2 and 293T, were purchased from The American Type Culture Collection (ATCC, USA). DMEM (Gibco, USA) with 10% FBS (Gibco, USA) and 1% penicillin/streptomycin (Gibco, USA) was used to culture cells at 37°C in a humidified atmosphere. The plasmids and short hairpin RNAs (shRNAs) used in this study were synthesized by and purchased from GenePharma (Shanghai, China). All transfections were conducted using lipofectamine 3000 or RNA iMax (Invitrogen, US) following the manufacturer’s instructions.

### Quantitative Real-Time PCR

Total RNA isolations were conducted using TRIzol Reagent (Invitrogen, US), and total cDNA was synthesized using the Superscript RT Kit (TOYOBO, Japan). Real-time PCR was performed using the SYBR Green PCR Master Mix Kit (TOYOBO, Japan). Endogenous glyceraldehyde 3-phosphate dehydrogenase (GAPDH) was used for normalization. The primers used in this study are as follows: LncRNA ASB16-AS1 forward: CGGCCCTGAGGCAAACATAC, reverse: TGAAACACTGCGCCAACTTC; miR-185-5p forward: CCATGTGCCTGTGTCATGC, reverse: ATCTGCTGATCCCCGCCA; miR-214-3p forward: ACACTCCAGCTGGGACAGCAGGCACAGACA, reverse: TGGTGTCGTGGAGTCG; LARP1 forward: GCAACCTAAAGACACTAC reverse: CCTCTTCTTCACTTCAATC; GAPDH forward: GCCTGCTTCACCACCTTCT, reverse: GAACGGGAAGCTCACTGG. The 2 -^ΔΔ^Ct method was used to calculate relative expression levels.

## Western Blot

Proteins from cells and tissues were extracted using Radioimmunoprecipitation (RIPA) lysis buffer (Beyotime, China). Protein concentration was determined using the bicinchoninic acid (BCA) kit (Beyotime, China). Next, protein samples were subjected to 10% SDS-PAGE and transferred to PVDF membranes. The membranes were blocked using 5% nonfat milk and washed three times in TBS with 0.1% Tween-20. The membranes were incubated with primary antibodies overnight at 4°C, followed by incubation with secondary antibodies for 1 h at room temperature. The primary antibodies used were; anti-LARP1 (1:1000, 13187S, CST), anti-E-cadherin (1:1,000, 14472S, CST), anti-Vimentin (1:1,000, 5741S, CST), and anti-GAPDH (1:5,000, ab8245, Abcam). The ECL Chemiluminescence System (Santa Cruz Biotechnology, US) was used to visualize antibody binding.

### 5‐Ethynyl‐2′‐Deoxyuridine (EdU) Incorporation

Cell proliferation was determined using the Cell-Light EdU DNA Cell proliferation kit (RiboBio, China) following the manufacturer’s instructions. Two days after transfection, 50 mM EdU was applied to cells an incubated for 2 h. DAPI was used to stain nucleic acids and Apollo Dye Solution used to stain cells. The cell proliferation rate was calculated using Image J software (NIH, USA).

### Cell Invasion and Migration Assays

Cell invasion and migration assays were performed in 24-well transwell plates filled with a polycarbonate membrane (pore size, 8 μm) (Corning, US), and Matrigel basement membrane matrix (1 μg/μl) (BD Biosciences, US) was used to fill the membranes. Briefly, 100 μl of serum-free media suspension was used to fill the upper chamber and 600 μl DMEM with 10% FBS was used to fill the lower chamber. After 24 h, the membranes of chambers were treated with crystal violet staining and observed under the microscope. Six fields of view were randomly chosen and cell numbers were recorded. Experiments were repeated three times.

### RNA Fluorescent *In Situ* Hybridization (FISH)

The FISH kit (Ribibio, China) was used to detect the location of ASB16-AS1 in the ccRCC cell line 786-O following the manufacturer’s protocol. Briefly, 786-O cells were incubated with pre-hybridization solutions for 30 min. Probes were treated with 20 μM hybridization solution and allowed to hybridize for 12 h. Next, saline sodium citrate was used to wash slides three times before treating with DAPI for 20 min. Results were visualized using a confocal microscope.

### Luciferase Reporter Assay

PmirGLO vectors (Promega, USA) harboring miR-185-5p and miR-214-3p sequences with wild-type or mutant binding sites for ASB16-AS1/LARP1 were used. The miR-185-5p mimic, miR-214-3p mimic, and the luciferase vectors were co-transfected into 786-O and 293T cells. The Dual-Luciferase Reporter Assay System (Promega, US) was used to detect luciferase activity.

### RNA Immunoprecipitation

Anti-AGO2 (#03-110, Millipore, Germany) was used to perform RNA immunoprecipitation (RIP) by using the Magna RIP RNA-binding protein immunoprecipitation kit (Millipore, Germany). To analyze the RNA bound complexes qRT-PCR assay was performed. Anti-IgG was used as an isotype control.

### RNA Pull-Down

Biotinylated ASB16-AS1, miR-185-5p, miR-214-3p, and control probes were synthesized and purchased from GenePharma (Shanghai, China). Co-immunoprecipitation buffer (Beyotime, China) was used to lyse cells which were then subjected to high amplitude. Cell lysates were incubated with ASB16-AS1, miR-185-5p, and miR-214-3p probe-streptavidin beads (Life, USA) overnight. TRIzol Reagent (Invitrogen, US) was used for RNA isolation and RNA bound complexes were analyzed by qRT-PC.

### *In Vivo* Mouse Xenografts

The Committee for Animal Care and Use of Shanghai general Hospital approved our animal experiments. Six-week old nonobese severe diabetic/severe combined immunodeficiency (NOD/SCID) mice were randomly divided into two groups (n=5 each). The ccRCC 786-O cells pre-transfected with Sh-NC or Sh-ASB16-AS1 were then subcutaneously inoculated into the NOD/SCID mice (1×10^7^ cells per tumor). From day 25 post inoculation, tumor volumes were measured every five days until day 45.

### Immunohistochemistry

After surgery, all specimens were collected and fixed in formalin immediately. Then, specimens were all subjected to the process of dehydration, paraffinization, and embedded in paraffin blocks. Subsequently, specimens were cut into sections at 4 µm and dried in air 12 h. Tissue sections were undergoing the process of dehydration, paraffinization once again, and subjected to antigen retrieval with sodium citrate buffer upon heat stimulation. Endogenous peroxidase activity was blocked by 3% hydrogen peroxide for 5 min. Next, tissue sections were incubated with primary antibody (LARP1; Abcam; 1:200; ab245635), and biotinylated goat anti-mouse IgG. Results were visualized using the VECTASTAIN ABC kit (Vector Laboratories) according to manufacturer’s protocol.

### Statistical Analysis

All experiments were carried out at least three times unless otherwise stated. Statistical analysis was performed using SPSS 19.0 (IBM, USA). The differences between two groups were tested using a Student’s t-test; whereas, a one-way ANOVA was used to analyze the difference between three or more group. Data are presented as mean ± SD, and P < 0.05 was considered as statistically significant.

## Results

### The Expression of ASB16-AS1 in ccRCC Tissues

**Figure 1:**
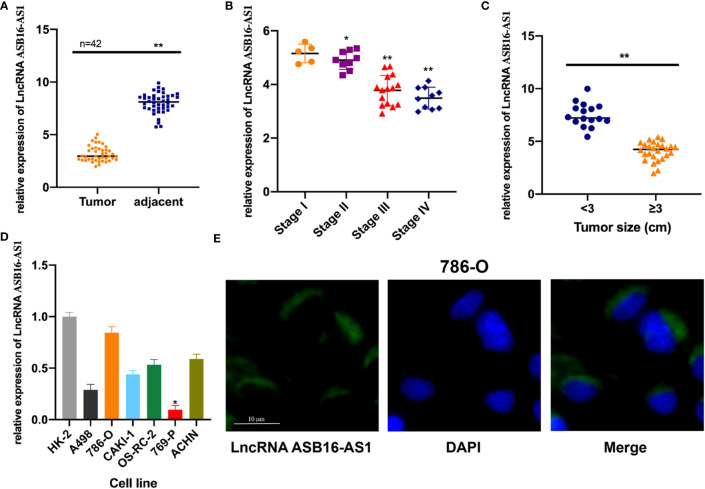
The expression of ASB16-AS1 in renal cell carcinoma (RCC) tissues. **(A)** qRT-PCR analysis showing decreased ASB16-AS1 expression in 42 RCC tumor tissues compared with paired adjacent non-diseased tissues. **(B)** ASB16-AS1 expression is significantly decreased in RCC tumors of higher stages (N=5–15). **(C)** ASB16-AS1expression is significantly decreased in larger tumors (N=16 smaller tumors and 26 larger tumors). **(D)** The expression of ASB16-AS1 varies across different RCC cell lines and the human kidney proximal tubular epithelial cell line, HK-2. **(E)** Fluorescent *in situ* hybridization showing the cytoplasmic location of ASB16-AS1 in 786-O cells. Scale bar – 10 μm. All experiments were repeated at least three times. *P < 0.05, **P < 0.01.

In order to investigate whether ASB16-AS1 plays a role in ccRCC progression, we tested ASB16-AS1 expression in 42 pairs of ccRCC tumor tissues and adjacent non-diseased tissues. We found that ASB16-AS1 expression in tumors was significantly lower than in adjacent non-diseased tissues (**Figure 1A**). Moreover, ASB16-AS1 was abundantly expressed in later stage and larger tumors (**Figures 1B, C**), suggesting that ASB16-AS1 might be involved in ccRCC initiation and progression. Next, we measured ASB16-AS1 expression in different ccRCC cell lines and the human proximal tubular epithelial cell line HK-2. The results showed that ASB16-AS1 was abundantly expressed in 786-O cells and lowly expressed in 769-P cells (**Figure 1D**). Fluorescent *in situ* hybridization (FISH) shows that ASB16-AS1 is mainly located in the cytoplasm of 786-O cells (**Figure 1E**).

### ASB16-AS1 Downregulation Promotes ccRCC Proliferation, Migration, and Invasion

**Figure 2:**
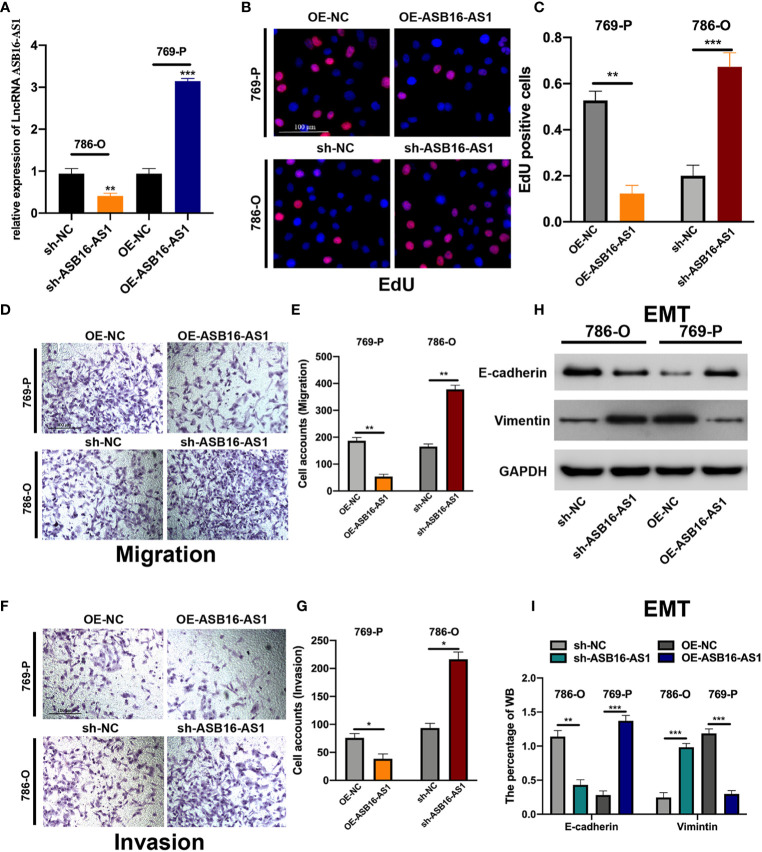
ASB16-AS1 downregulation promotes renal cell carcinoma (RCC) proliferation, migration, and invasion. **(A)** The transfection efficiency of ASB16-AS1 siRNA/NC and OE/NC in 786-O and 769-P cells. **(B)** Representative images of EdU positive cells showing proliferation of shRNA treated 786-O and 769P cells, which are quantified in **(C)**. **(D)** Representative images of the transwell migration assays used to detect the migration of shRNA treated 786-O and 769P cells, which are quantified in **(E)**. **(F)** Transwell invasion assays were applied to detect the invasion of shRNA treated 786-O and 769P cells, which are quantified in (G). **(H)** Western blots of epithelial-mesenchymal-transition associated genes in shRNA treated 786-O and 769P cells, which are quantified in **(I)**. All experiments were repeated at least three times, scale bars -100 μm. *P < 0.05, **P < 0.01, ***P < 0.001.

Next, we determined the biological function of ASB16-AS1 in ccRCC progression. Cell models were generated by transfecting sh-NC or sh-ASB16-AS1 into 786-O cells, and OE-NC or OE-ASB16-AS1 into 769-P cells, respectively. Transfection efficiency is shown in **Figure 2A**. Cell proliferation was determined by EdU assays. As shown in **Figures 2B, C**, downregulation of ASB16-AS1 promoted cell proliferation in 786-O cells, and upregulation of ASB16-AS1 inhibited cell proliferation in 769-P cells. Cell migration and invasion was detected by performing Transwell assays. It was found that downregulation of ASB16-AS1 promoted migration (**Figures 2D, E**) and invasion (**Figures 2F, G**) in 786-O cells, and upregulation of ASB16-AS1 inhibited migration (**Figures 2D, E**) and invasion (**Figures 2F, G**) in 769-P cells. Next, epithelial-mesenchymal transition (EMT) was investigated by measuring E-cadherin and Vimentin protein expression in treated cells. As shown in **Figures 2H, I**, ASB16-AS1 overexpression promoted an EMT phenotype in 769-P cells, and ASB16-AS1 downregulation inhibited an EMT phenotype in 786-O cells. These results indicate that ASB16-AS1 is involved in ccRCC progression.

### ASB16-AS1 Downregulation Promotes ccRCC Cell Growth and Metastasis *In Vivo*

**Figure 3:**
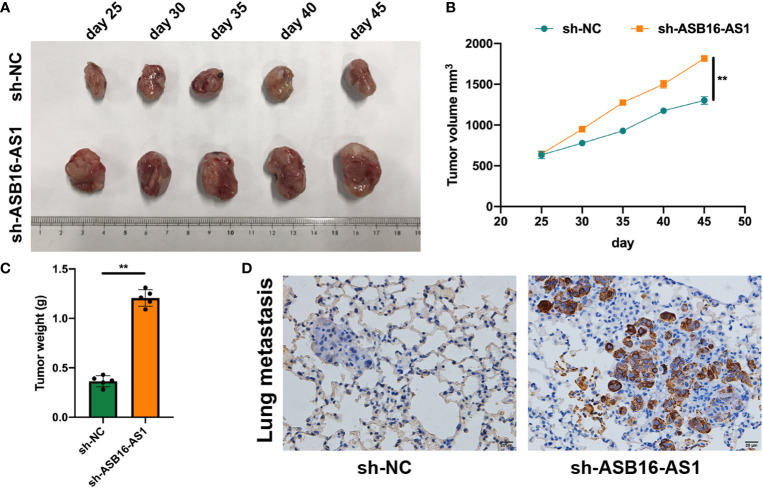
ASB16-AS1 downregulation promotes renal cell carcinoma (RCC) cell growth and metastasis *in vivo*. Nonobese severe diabetic/severe combined immunodeficiency mice (6 weeks old) were subcutaneously inoculated with 786-O cells (1×10^7^ per tumor) pre-transfected with Sh-NC or Sh-ASB16-AS1 (N=5 per condition). **(A)** Representative images of inoculated tumors which are quantified by volume in **(B)** which shows tumor volume increasing over time in the Sh-ASB16-AS1 pre-treated cell condition. **(C)** Final tumor weights show that Sh-ASB16-AS1 pre-treated cells produces heavier tumors (N=5 per condition). **(D)** Representative histological images of lung metastasis. Data are presented as mean ± S.D, scale bars – 20 μm. **P < 0.01.

*In vivo* experiments were applied to further assess the biological functions of ASB16-AS1 in ccRCC progression. Nonobese severe diabetic/severe combined immunodeficiency (NOD/SCID) mice (6 weeks old) were subcutaneously inoculated with 786-O cells (1×10^7^ per tumor) which were pre-transfected with sh-NC or sh-ASB16-AS1. The representative images of excised tumors are shown in **Figure 3A**. From day 25 post inoculation, tumor volumes were recorded every 5 days until day 45 (**Figure 3B**), comparative statistics of final tumor weights are shown in **Figure 3C**. it was suggested that downregulation of ASB16-AS1 significantly inhibits tumor growth compared with control levels of ASB16-AS1. Lung tissues from xenografted mice using hematoxylin and eosin staining showed that downregulation of ASB16-AS1 enhanced tumor metastasis (**Figure 3D**).

### ASB16-AS1 Sponges miR-185-5p and miR-214-3p

**Figure 4:**
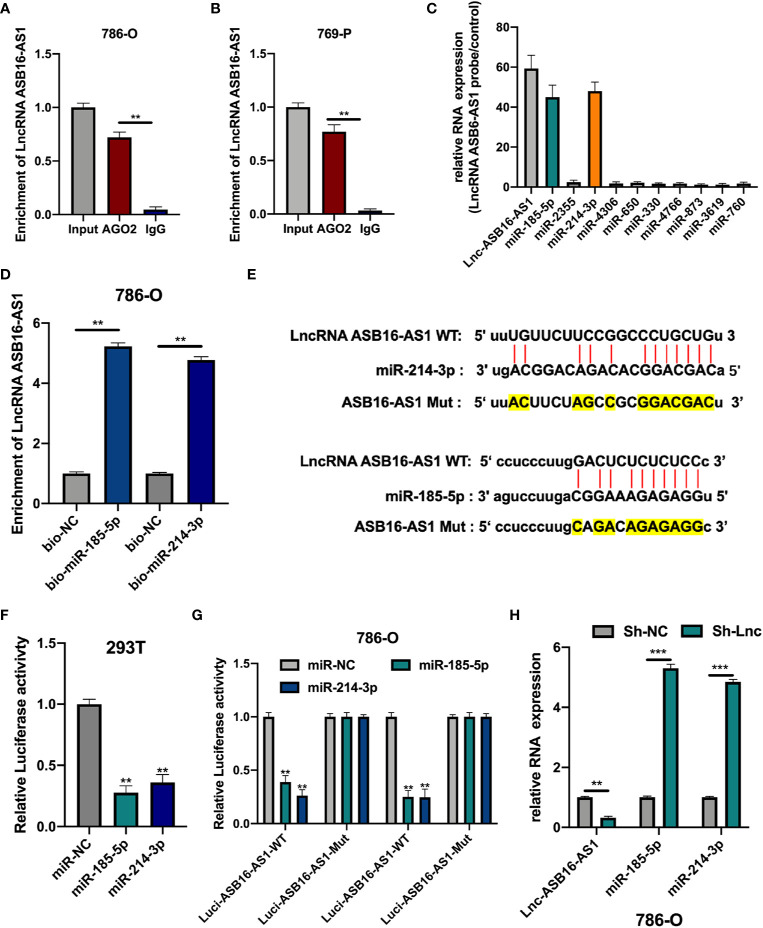
ASB16-AS1 sponges miR-185-5p and miR-214-3p. **(A, B)** AGO2-RIP experiments were conducted to detect the potential miRNA binding ability of ASB16-AS1 by detecting the binding of ASB16-AS1 to the AGO2 protein in 786-O and 769-P cells, respectively. **(C)** The expression of potential miRNA targets in ASB16-AS1 probe RNA bound complexes (according to the number of supported AGO CLIP-seq experiments), measured by qRT-PCR and normalized to the control probe in 786-O cells. **(D)** Biotinylated RNA pull-downs were conducted to measure the enrichment of ASB16-AS1 in biotinylated miR-185-5p and miR-214-3p conditions. **(E)** Wild-type (WT) and mutated (MUT) sequences of the putative ASB16-AS1 miR binding sites aligned with miR-185-5p and miR-214-3p. Mutated bases are highlighted in yellow. **(F, G)** Luciferase reporter activity of Luc-ASB16-AS1-WT or MUT in 293T **(F)** and 786-O cells **(G)** after transfection with miR-185-5p and miR-214-3p. **(H)** relative RNA expression in Sh-NC and Sh-ASB16-AS1 transfected 786-O cells, measured by qRT-PCR. All experiments were repeated at least three times. **P < 0.01, ***P < 0.001.

Since lncRNA ASB16-AS1 is involved in ccRCC progression both *in vitro* and *in vivo* we sought to further understand the role of ASB16-AS1; therefore, we investigated its underlying molecular mechanisms. AGO2-RIP experiments were performed to assess the miRNA binding ability of ASB16-AS1. As shown in **Figures 4A, B**, compared with the anti-IgG group, ASB16-AS1 was significantly enriched in anti-AGO2 complexes in 786-O and 769-P cells. Next, we conducted bioinformatics analysis using the Miranda program (http://www.microrna.org/microrna/home.do). The expression of selected miRNAs was measured in 786-O cells and normalized to a control probe. We found that miR-185-5p and miR-214-3p were abundantly expressed (**Figure 4C**). Next, RNA pull-down assays using bio-miR-185-5p and bio-214-3p probes were performed. ASB16-AS1 was highly enriched in bio-miR-185-5p and bio-214-3p RNA complexes compared with bio-NC (**Figure 4D**). Subsequently, we constructed wild type (WT) and mutant (Mut) ASB16-AS1 binding sites for miR-185-5p and miR-214-3p, respectively (**Figure 4E**). Luciferase activity assays were conducted as shown in **Figures 4F, G**; the luciferase activity of the ASB16-AS1 WT sequence significantly reduced when co-transfected with miR-185-5p or miR-214-3p mimic in 293T and 786-O cells. The expression of miR-185-5p and miR-214-3p in Sh-ASB16-AS1 transfected 786-O cells was significantly greater than in the control group (**Figure 4H**). These data suggest that ASB16-AS1 interacted with miR-185-5p and miR-214-3p.

### MiR-185-5p and miR-214-3p Inhibitors Rescue the Effects of Downregulated ASB16-AS1 on ccRCC Progression

**Figure 5:**
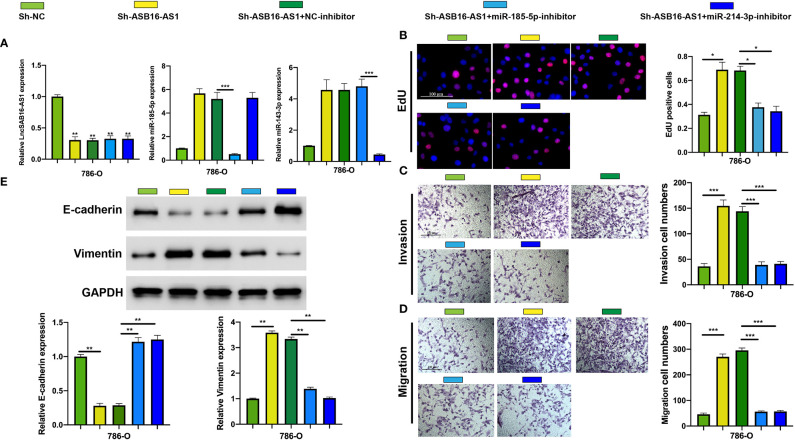
MiR-185-5p and miR-214-3p inhibition rescues the promotive effects of downregulated ASB16-AS1 on HCC progression. **(A)** Cell models were generated by transfecting Sh-NC, Sh-ASB16-AS1, Sh-ASB16-AS1+NC-inhibitor, Sh-ASB16-AS1+miR-185-5p-inhibitor, and Sh-ASB16-AS1+miR-214-3p-inhibitor in 786-O cells, respectively. The expression of ASB16-AS1, miR-185-5p, and miR-214-3p in shRNAs and inhibitor conditions were measured by qRT-PCR. **(B)** Determination of cell proliferation ability by performing EdU assays. **(C)** Determination of cell invasion ability by using Transwell invasion assays. **(D)** Determination of cell migration ability by using Transwell migration assays. **(E)** Expression of the epithelial-mesenchymal-transition associated proteins E-cadherin and Vimentin were detected by western blot assays, which are quantified in the bottom left graphs. All experiments were repeated at least three times. Scale bars -100 μm, *P < 0.05, **P < 0.01, ***P < 0.001.

To further demonstrate the association between ASB16-AS1 and miR-185-5p/miR-214-3p, and their biological functions in ccRCC, we generated cell models by transfecting 786-O cells with Sh-NC, Sh-ASB16-AS1, Sh-ASB16-AS1+NC-inhibitor, Sh-ASB16-AS1+miR-185-5p-inhibitor, and Sh-ASB16-AS1+miR-214-4p inhibitor. Transfection efficiencies are shown in **Figure 5A**. To assess the biological functions of treated 786-O cells, EdU, and Transwell assays were conducted. The promotive effects of downregulated ASB16-AS1 on cell proliferation (**Figure 5B**), cell invasion (**Figure 5C**), and cell migration (**Figure 5D**) were rescued by the miR-185-5p and miR-214-3p inhibitors. Furthermore, miR-185-5p and miR-214-3p inhibition effectively alleviated the enhancement of downregulated ASB16-AS1 on the EMT phenotype (**Figure 5E**). These data indicate that ASB16-AS1 regulates ccRCC progression *via* miR-185-5p and miR-214-3p.

### LARP1 Is a Downstream Target for miR-185-5p/miR-214-3p

**Figure 6:**
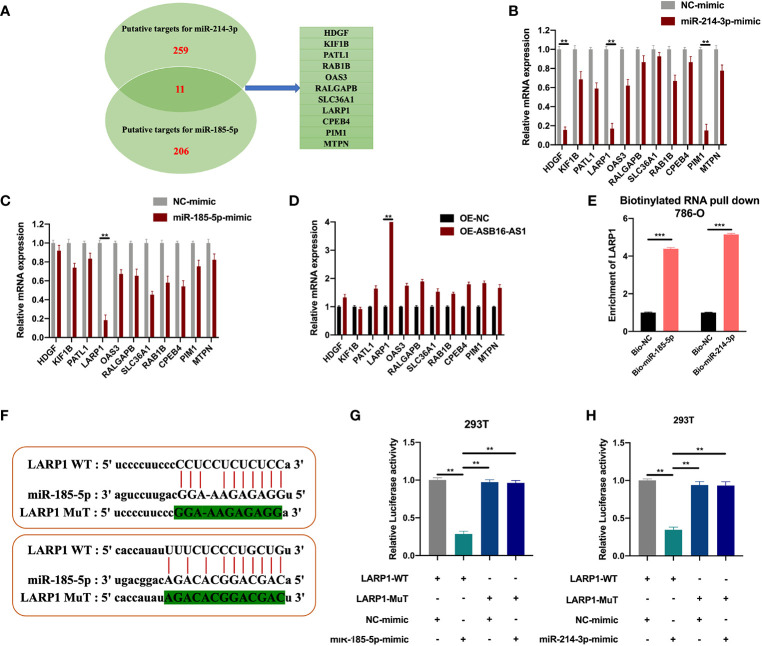
LARP1 is a downstream target for miR-185-5p/miR-214-3p. **(A)** The downstream targets of miR-185-5p and miR-214-3p were predicted using the ENCORI database (http://starbase.sysu.edu.cn/index.php), with CLIP Data; high stringency (>=3) and Predicted program (microT, miRanda, miRmap, and PITA). Eleven potential mRNA targets were chosen. 786-O cells were transfected with NC-mimic/miR-185-5p mimic, NC-mimic/miR-214-3p, and OE-NC/OE-ASB16-AS1, respectively. **(B–D)** Relative mRNA expression of potential target mRNAs, measured by qRT-PCR. **(E)** Biotinylated RNA pull-down assays were performed and qRT-PCR experiments were conducted to determine the enrichment of LARP1 in Bio-NC/Bio-miR-185-5p, Bio-NC/Bio-miR-214-3p treated 786-O cells. **(F)** Wild-type (WT) and mutated (MUT) sequences of the putative LARP1 miR binding sites aligned with miR-185-5p, and miR-214-3p. **(G, H)** Luciferase reporter activity of Luc-LARP1-WT or MUT in 293T **(G)** and 786-O cells **(H)** after transfection with miR-185-5p and miR-214-3p mimics and NC-mimic. All experiments were repeated at least three times. **P < 0.01, ***P < 0.001.

The downstream targets of miR-185-5p and miR-214-3p were predicted using the ENCORI database (http://starbase.sysu.edu.cn/index.php). Eleven putative targets of miR-185-5p and miR-214-3p were selected (**Figure 6A**), and measured in NC-mimic/miR-185-5p-mimic, NC-mimic/miR-214-3p-mimic, and OE-NC/OE-ASB16-AS1 transfected 786-O cells, respectively. It was found that miR-185-5p and miR-214-3p significantly suppressed La-related protein 1 (LARP1) expression (**Figures 6B, C**), results of the expression of HDGF and PIM1 NC-mimic/miR-214-3p-mimic transfected 786-O cells were in accordance with previous studies (21, 22). However, LARP1 was effectively upregulated upon ASB16-AS1 overexpression (**Figure 6D**). Therefore, we speculated that LARP1 might interact with miR-185-5p and miR-214-3p. Biotinylated RNA pull-downs using bio-miR-185-5p or bio-miR-214-3p were performed. As shown in **Figure 6E**, LARP1 was significantly enriched in bio-miR-185-5p and bio-214-3p RNA complexes compared with bio-NC. Next, The LARP1 WT and MuT sequences targeted to miR-185-5p and miR-214-3p binding sites were constructed (**Figure 6F**). The LARP1 WT sequence effectively reduced luciferase activity when co-transfected with a miR-185-5p or miR-214-3p mimic in 293T cells (**Figures 6G, H**), demonstrating that LARP1 interacts with both miR-185-5p and miR-214-3p.

### Downregulated ASB16-AS1 Promotes ccRCC Progression *Via* the miR-185-5p/miR-214-3p-LARP1 Pathway

**Figure 7:**
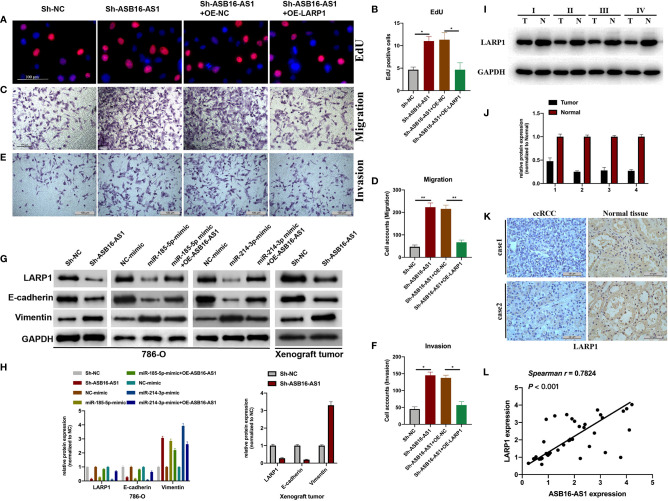
Downregulated ASB16-AS1 promotes ccRCC progression *via* the miR-185-5p/miR-214-3p-LARP1 pathway. 786-O cells were stably transfected with Sh-NC, Sh-ASB16-AS1, Sh-ASB16-AS1+OE-NC, and Sh-ASB16-AS1+OE-LARP1. **(A, B)** Determination of cell proliferation using EdU assays (Scale bars -100 μm). **(C, D)** Determination of cell invasion ability using Transwell migration assays (Scale bars -100 μm). **(E, F)** Determination of cell migration ability using Transwell invasion assays (Scale bars -100 μm). **(G, H)** Expression of the epithelial-mesenchymal-transition associated proteins E-cadherin and Vimentin were detected by western blot assays. **(I, J)** Expression of LARP1 in four ccRCC tumor tissues and its adjacent normal tissues, which were randomly selected, were detected using western-blot. **(K)** Expression of LARP1 in two ccRCC tumor tissues and its adjacent normal tissues, which were randomly selected, were assessed using IHC assay (Scale bars -50 μm). **(L)** The expression correlation between ASB16-AS1 and LARP1 were analyzed using spearman statics. *P < 0.05, **P < 0.01.

Next, we assessed the role of LARP1 upon ASB16-AS1 downregulation. Firstly, EdU and Transwell assays were performed. As shown in **Figures 7A–F**, the promotive effects of downregulated ASB16-AS1 on cell proliferation (**Figures 7A, B**), cell invasion (**Figures 7C, D**), and cell migration (**Figures 7E, F**) were rescued by LARP1 overexpression. Moreover, LARP1 overexpression effectively alleviated the enhancement of downregulated ASB16-AS1 on the EMT phenotype both in 786-O cells and xenograft tumor tissues (**Figures 7G, H**). The expression of LARP1 significantly decreased in ccRCC tumor tissues compared with its adjacent normal tissues (**Figures 7I–K**). Spearman statics results suggested that the expression of ASB16-AS1 was strongly correlated with LARP1 expression in ccRCC tumor tissues. Our findings suggested that downregulated ASB16-AS1 play its promotive effect on ccRCC progression through miR-185-5p/miR-214-3p-LARP1 pathway.

## Discussion

Despite the improvement of RCC clinical management in the past decades, RCC still is the sixth most frequently diagnosed cancer in men and tenth in women, which has been a great threaten to people health (23). As one most lethal pathological subtype of RCC, the diagnosis and intervention of ccRCC present great challenge to urologist. While, its underlying mechanisms still remain unclear. The clinical intervention of ccRCC demands novel targets more than ever.

In the current study, our results demonstrate the role of ASB16-AS1 in ccRCC progression. By performing *in vitro* and *in vivo* experiments, we found that ASB16-AS1 expression was downregulated in ccRCC tissues, which was also associated with a later tumor stage and larger tumors. Subsequently, our findings demonstrated the biological functions of ASB16-AS1 in ccRCC progression. Downregulation of ASB16-AS1 promoted cell proliferation, migration, and invasion, regulated EMT associated genes in ccRCC cells and promoted tumor growth and metastasis in a xenograft mouse model. Furthermore, it was found that ASB16-AS interacts with miR-185-5p and miR-214-3p. It has been reported that miR-185-5p is involved in cancer development and regulates tumorigeneses *via* its involvement in cell proliferation, migration, invasion, and apoptosis (24–26). Additionally, miR-214-3p is involved in osteosarcoma, breast cancer, endometrial cancer, and lung cancer (27–30). These studies suggest that miR-185-5p and miR-214-3p play important roles in tumorigeneses and tumor development. However, neither have been studied in ccRCC.

Here, our findings suggested that ASB16-AS1 acted as a molecular sponge for miR-185-5p and miR-214-3p. Furthermore, the promotive effects of ASB16-AS1 on cell proliferation, migration, invasion, and EMT associated gene expression were rescued by miR-185-5p and miR-214-3p inhibition. While, the relationship between miR-185-5p and miR-214-3p, and their contribution in ccRCC cellular progression upon ASB16-AS1 downregulation still need further exploration. Moreover, the downstream transcriptional targets of ASB16-AS1 were not the final answer for its role in ccRCC biological progression. Therefore, bioinformatic tools, biotinylated RNA pull-down and luciferase reporter assays were used to identify its post-transcriptional target. Our findings found that LARP1 was targeted by miR-185-5p and miR-214-3p in 786-O and 293T cells.

LARP1, as one RNA binding protein, has been in-depth studied recently due to its capability to interact with mammalian target of rapamycin complex 1 (mTORC1) and act as a key repressor of ribosomal protein mRNA translation (31–34). The biological role of LARP1 was investigated in non-small cell lung cancer, ovarian cancer, and hepatocellular carcinoma (35–37). While, the molecular mechanisms of LARP1 in ccRCC progression still uncovered. Since the crucial role of LARP1 in ribosome production, which is an essential unit for cellular progression in all living organisms (38). We presumed that the biological effects of ASB16-AS1/miR-185-5p/miR-214-3p in ccRCC progression were functioned through regulating LARP1. Our results showed that overexpression of LARP1 reversed the promotive effects of downregulated ASB16-AS1 on ccRCC cellular progression and EMT phenotype. Furthermore, the expression ASB16-AS1 and LARP1 in ccRCC tumor tissues were suggested strongly correlated. Although, the role of ASB16-AS1/miR-185-5p/miR-214-3p/LARP1 pathway in ccRCC progression has been partially demonstrated. While, in the contrast with previous findings, our results implicated that LARP1 exerts its tumor suppressive effect in ccRCC cellular progression, which might owe to the timepoint-dependent feature of LARP1 knockdown (39). Furthermore, since the role of mTOR-LARP1 axis in cancer cellular progression is universally agreed, we assumed that LARP1 might exert its function in ccRCC through this axis, which requires in-depth investigation in our future study.

## Conclusion

In the current study, we demonstrate the interaction of miR-185-5p and miR-214-3p with LARP1 and show the role of the ASB16-AS1-miR-185-5p/miR-214-3p-LARP1 pathway in ccRCC progression. Collectively, our data determined the role of ASB16-AS1, miR-185-5p, and miR-214-3p in ccRCC progression. Moreover, we identified a mechanism of upstream regulation of LARP1 in ccRCC. We therefore provide new insights into ccRCC basic research, and present potential prognostic and therapeutic targets for ccRCC clinical management.

## Data Availability Statement

The data used to support the findings of this study are available from the corresponding author upon request.

## Ethics Statement

The animal study was reviewed and approved by Shanghai General Hospital, School of Medicine, Shanghai Jiaotong University. Written informed consent was obtained from the individual(s) for the publication of any potentially identifiable images or data included in this article.

## Author Contributions

JF, WL, and JX designed the research. ML, BY, and MC performed the research and wrote the manuscript. JP, ZD, and XM helped in the analysis of the data. All authors contributed to the article and approved the submitted version.

## Funding

This work was supported by the Youth Program of National Natural Science Foundation of China (81803013 to JP),

## Conflict of Interest

The authors declare that the research was conducted in the absence of any commercial or financial relationships that could be construed as a potential conflict of interest.

## Abbreviations

AGO2, argonaute RISC catalytic component 2; ASB16-AS1, ASB16 antisense RNA 1; ccRCC, clear cell renal cell carcinoma; EdU, 5‐ethynyl‐2′‐deoxyuridine; EMT, epithelial-mesenchymal transition; IHC, immunohistochemistry; LARP1, La-related protein 1; LncRNA, long noncoding RNA; MiR, microRNA; NOD/SCID, nonobese severe diabetic/severe combined immunodeficiency mice; RCC, Renal cell carcinoma; RIP, RNA binding protein immunoprecipitation; shRNA, short hairpin RNA.
